# Literature Review Concerning the Challenges of Implementing Pharmacogenetics in Primary Care Practice

**DOI:** 10.7759/cureus.9616

**Published:** 2020-08-08

**Authors:** Mohamed S Omer

**Affiliations:** 1 Pharmacology, The Ohio State University College of Pharmacy, Columbus, USA

**Keywords:** pathology and clinical biochemistry, pharmacogenetics, genetics and molecular biology, outpatient family medicine, psychiatry & mental health

## Abstract

Since President Obama signed the Precision Medicine Initiative in 2015, endeavors to integrate pharmacogenetics in clinical practice and psychiatric care have been evolving rapidly. The nature of general practice and psychiatric medicine, including psychopharmacotherapy and the long-term care necessary for chronic diseases, renders these fields in desperate need of the implementation of pharmacogenetics. This article presents some of the challenges facing pharmacogenetics implementation in family medicine and psychiatric care. Reputable research websites were used to extract papers, data, and lectures concerning this topic. The results reveal that three main challenges are facing this integration: the evaluation of pharmacogenetic testing in general and psychiatric practice, cost-effectiveness, and regulatory burdens. Although considerable advances are being made to address these issues, it is time to gather these efforts under one umbrella to create guidelines based on previous and upcoming research.

## Introduction and background

Primary care is constantly evolving. It is complex, and massive portions of it remain untapped. General practitioners and scientists in the psychiatry, neurology, and neuroscience fields are rushing to discover the pathophysiology of many chronic diseases and psychiatric disorders. However, sometimes discoveries of an illness’s pathophysiology can stagnate or retrograde after a drug has been developed to successfully cure the disorder. Such instances require enormous amounts of data at the genetic and molecular level to precisely determine the location and the mutation variations related to diseases among different populations, so treatment may be applied at the individual level. Considering that most chronic diseases and psychiatric disorders usually require long-term medication compliance, patients are subjected to the adverse events that accompany pharmacotherapy. At this point, the importance of using the current genomic data to locate a biomarker for present and upcoming medications becomes evident. Implementation of this approach in primary care will allow clinicians to tailor pharmacotherapy to the needs of the patient and determine whether that patient will be subjected to adverse events. Although great advancements and breakthroughs have been made in the pharmacogenetics field, its integration in these domains faces many challenges, such as the validity of specific biomarkers, the cost of procedures, regulatory hurdles, acceptance among healthcare teams, and whether the pharmacoeconomic benefits from these procedures outweigh the benefits of abstaining from them.

## Review

A literature search and review were conducted using literature screening through various research websites, including the Ohio State University Library, PubMed, Online Mendelian Inheritance in Man (OMIM), the Human Genome Organisation (HUGO) Gene Nomenclature Committee (HGNC), and Google Scholar.

This issue may be categorized in a considerable number of ways; however, the present author believes the categorization constructed by de Leon et al. is the most comprehensive, reflecting the challenges facing the enforcement of pharmacogenetic testing in general practice and psychiatric care [1]. The author has, therefore, used the same categorization but conducted an extensive search for other studies pertaining to each category.

Evaluation of pharmacogenetic testing in primary care practice

Implementing pharmacogenetic testing in primary care is promising. However, pharmaceutical companies strive to validate pharmacogenetic tests following the same pattern they use for drug development, which means the tests must successfully pass from the preclinical phase to Phase IV post-marketing surveillance studies [1]. This approach is not practical for pharmacogenetic tests because validating them is more complicated than developing new drugs. The pharmacogenetic validation process is required, not only to prove its validity through traditional sensitivity and specificity measures, but also to attain sensitivity and specificity at three levels: (1) identifying mutations versus non-mutations, (2) detecting different phenotypes, and (3) confirming that a phenotype conveys a clinical case [1].

Cost-effectiveness

This is one of the most debatable points of pharmacogenetic implementation. Though extensive pharmacoeconomic studies are necessary, some researchers have already published papers concerning drugs used in psychiatric practice. One study points out the cost-effectiveness of the pharmacogenetics test associated with clozapine [2]. The study examines these metrics in relation to administering clozapine as a first-line drug for patients whose test results reveal they have a genetic predisposition for responding well to it, versus administering it as a third-line drug. The authors find no significant difference in quality-adjusted life-years (QALYs). Furthermore, there is an additional cost of about US $47,705 [2]. The same study compares the impact of the test’s cost-effectiveness with its sensitivity and specificity, concluding that cost-effectiveness highly significantly influences whether this test is used [2].

However, another study analyzing the cost-effectiveness of introducing a pharmacogenetic test (NeuroIDgenetix) in treatment plans for major depression provides a different perspective [3]. It mentions that, in 2016, the total per-patient economic burden of major depressive disorder was US $34,585. After integrating the pharmacogenetic test in treatment plans, the charge reduced to US $27,099, which saved the economy approximately US $7,486 [3].

A third interesting article considers a crucial point: the cost-effectiveness of using combinatorial pharmacogenomics instead of single gene detection. The author claims that using single gene mutation detection as a pharmacogenetic test for psychiatric disorders has yielded unsatisfactory results; therefore, using combinatorial gene mutation detection is more beneficial. For example, the article cites that implementing combinatorial pharmacogenetic tests in patients with treatment-resistant major depressive disorder increases QALYs by 0.316 years, which, in return, reduces the direct medical cost for an average of US $3,711 [4].

Regulatory oversight

The argument concerning the regulatory disputes that face the implementation of pharmacogenetic testing in primary care is branched and complicated. It discusses the development of a unified regulatory framework, which would govern the safety and efficacy of pharmacogenetic testing integration. The regulatory issues facing pharmacogenetics in general and psychiatric practices are divided into three main points: analytical validation, qualification, and clinical utility.

The analytical validation of a pharmacogenetic test examines its ability to detect biomarkers or genetic variants in a reproducible and trusted pattern. This should include a test’s ability to produce reliable results in terms of analytical performance, which represents precision, accuracy, and strength for detecting relevant phenotypes [5].

Based on this, the importance of the combinatorial pharmacogenetic test arises again, as it can satisfy the requirements of analytical validation. The combinatorial test consists of two parts: evaluating the genotype of each variant and interpreting the common genotypes. A study researching the combinatorial test has demonstrated that the reproducibility of a group of combinatorial pharmacogenetic tests for neuropsychiatric disorders is more than 99.9%. The accuracy of the combinatorial test has also reached 99.8%. That study, therefore, concludes that it is critical to incorporate the combinatorial pharmacogenetic test in decision-making for treating neuropsychiatric disorders, especially those with extreme phenotypes [5].

Qualification measures a test’s tendency to determine the disorder in question. To date, little information has been extrapolated from scientific research targeting this issue. However, some studies have investigated the clinical validity of using the pharmacogenetic test in decision analysis for treating major depressive disorder. One group of authors uses GeneSight Psychotropic as a combinatorial pharmacogenetic testing tool. They divide the results into three colors: green, yellow, and red based on the prescription advisory [6]. Green color represents “Use as Directed”, yellow color represents “use with Caution”, and red represents “Use with Very Caution”. However, if a doctor does conduct a pharmacogenetic test, it is highly likely that the test will show whether the patient will suffer from drug-drug interactions, drug side effects, or underestimated treatment results, and this indicates that pharmacogenetic testing has a highly beneficial clinical validity when applied to major depressive disorder [6].

Clinical utility, the third regulatory debate, is defined as a test’s ability to refine the risks and benefits of its use. Essentially, this point summarizes the whole previous argument for implementing pharmacogenetic tests in psychiatric practice. A study concerning the clinical utility of these tests summarizes the numerous benefits of integrating them in primary care, such as determining the precise outcomes for prescribed medications and avoiding adverse events that might occur because of patients’ varying metabolisms. However, with advancements in the drug development process, scientists and manufacturers can now more sophisticatedly produce neuropsychiatric medication, which is safer than previous generations of medication. This, in turn, may make medical teams reluctant to order these tests to avoid the financial burden associated with them; some may lean toward prescribing medications based on current guidelines and postponing ordering pharmacogenetic tests until there is some evidence for doing so. Other physicians may prefer, especially in mild to moderate cases, to prescribe medication directly based on their experiences with these kinds of drugs [7].

 

Discussion

As evidenced above, many challenges are associated with integrating pharmacogenetics into family medicine and psychiatric practice. Although the value of this implementation is becoming increasingly evident, more effort is required to prove its validity when applied to most chronic diseases and psychiatric disorders. Unfortunately, at present, only a few studies have been conducted with significant disorders. However, with the abundance of genetic information, efforts must be directed toward more validation research.

One of the most influential factors influencing the use of pharmacogenetics in primary care practice is the cost of implementing the tests. Several factors must be considered when analyzing the cost-effectiveness of pharmacogenetic implementation. Some directly relate to the diseases, such as mortality rate, cost of inpatient treatment, cost of outpatient treatment, and acuteness of the case. Other factors relate to the drugs, such as cost, effectiveness for treating each case, and the seriousness of drug-associated adverse events. The third group of factors is associated with the test itself. These factors include cost, accuracy, and therapeutic outcomes. Given the decreasing costs of genetic testing and its increasing availability, the present author has looked ahead to a possible future where genotype information might be readily available, at negligible cost, for all patients as part of their electronic health records.

Another point of discussion involves regulatory affairs. Pharmacogenetics testing, in general, has passed the introductory level of regulatory issues, meaning that it is fully recognized among regulatory bodies and in the medical communities. Now, pharmacogenetic testing is at the level of validation, especially for psychiatric care. However, the considerable efforts in this process are quite distracted and separated or are focused on a specific disease or group of disorders. Therefore, it would be more effective if these efforts were unified under one regulatory body to direct them toward formulating factual guidelines.

## Conclusions

Given the decreasing costs of genetic testing and its increasing availability, the present author has looked ahead to a possible future where genotype information might be readily available, at negligible cost, for all patients as part of their electronic health records. However, at present, pharmacologists, pharmacoepidemiologists, general practitioners, psychiatrists, clinical psychologists, pharmacogenetic scientists, and regulatory scientists must collaborate to integrate pharmacogenetics into family medicine and psychiatric care. This collaboration demands organization and structure to produce guidelines that regulate implementation. These guidelines must address the test’s pharmacoeconomic utility, qualification, and clinical utility. The guidelines should also be designed comprehensively, not only to lead clinicians, but also to create consensus between clinical teams and third-party payers, such as healthcare insurance companies and drug development companies.
